# A pilot study on transient ischemic stroke induced with endothelin-1 in the rhesus monkeys

**DOI:** 10.1038/srep45097

**Published:** 2017-03-30

**Authors:** PeiMin Dai, Hui Huang, Lin Zhang, Jing He, XuDong Zhao, FuHan Yang, Ning Zhao, JianZhen Yang, LongJiao Ge, Yu Lin, HuaLin Yu, JianHong Wang

**Affiliations:** 1 Key Laboratory of Animal Models and Human Disease Mechanisms of the Chinese Academy of Sciences & Yunnan Province, Kunming Primates Research Center, Kunming Institute of Zoology, Chinese Academy of Sciences, Kunming, China; 2 Department of Neurosurgery, First People’s Hospital of Honghe State, Honghe, China; 3 Department of Neurosurgery, Second Affiliated Hospital of Nanchang University, Nanchang, China; 4 Department of Neurosurgery, Center People’s Hospital of Zhumadian State, Zhumadian, China; 5 Second Department of Neurosurgery, First Affiliated Hospital of Kunming Medical University, Kunming, China; 6 Yunnan Key Laboratory of Primate Biomedical Research Institute of Primate Translational Medicine, Kunming University of Science and Technology, Kunming, China; 7 State Key Laboratory of Brain and Cognitive Science, Institute of Biophysics, Chinese Academy of Sciences, Beijing, China

## Abstract

Endothelin-1 (ET-1), a vasoconstrictor, has recently been used to induce focal ischemia in rodents and marmoset monkeys. The rhesus monkey, however, has numerous advantages to the rodent and marmoset that make it a superior and irreplaceable animal model for studying stroke in the brain. In the present study, after mapping the preferred hand representation in two healthy male monkeys with intracortical micro-stimulation, ET-1 was microinjected into the contralateral motor cortex (M1) to its preferred hand. The monkeys had been trained in three manual dexterity tasks before the microinjection and were tested for these tasks following the ET-1 injection. Brain Magnetic Resonance Imaging scans were performed 1, 7, 14 and 28 days post ischemia. It was found that ET-1 impaired the manual dexterity of the monkeys in the vertical slot and rotating Brinkman board tasks 3–8 days after the injection. Brain imaging found that severe edema was present 7 days after the focal ischemia. This data suggest that ET-1 can induce transient ischemic stroke in rhesus monkey and that ET-1 induced focal ischemia in non-human primates is a potential model to study the mechanism of stroke and brain repair after stroke.

Ischemic stroke in humans is the second leading cause of death and disability worldwide^1^. Survivors are likely to sustain lifelong impairments dependent on the size and localization of the brain injury that can affect sensory, motor, cognitive, behavioral, communicative and/or emotional functioning^2^. Future research into neuroprotective therapies against brain injury from strokes will benefit from an efficient animal model targeted toward focal ischemia.

At present, animal models of cerebral ischemia have mainly been developed in rodents^3^,^4^. However, the differences between humans and rodents—particularly in terms of genetics, pathology and pharmacology— significantly limits the use of rodent models of stroke in the development of neuroprotective therapies. Often, experimental neuroprotective therapies discovered and tested in rodents fail to make it to clinical use^5^. Non-human primates, in comparison to rodents, are more closely related to humans in terms of taxonomic status and possess a sophisticated and developed prefrontal cortex (PFC) similar to humans. As such, non-human primates have numerous intrinsic advantages over rodents that make them an irreplaceable animal model for studying cerebral ischemia that may lead to the development of more effective and/or efficient therapies to treat or protect against human strokes.

Endothelin-1 (ET-1), a 21-amimo acid peptide with potent and long-acting vasoconstriction properties, was first identified as an endothelium-derived contracting factor by Yanagisawa’s group in 1988^6^. ET-1 has recently been used to induce focal ischemia leading to impaired executive memory function and to impaired pure-motor and sensorimotor behaviors that are dependent on the specific area of ischemic insult in rodents^7^,^8^,^9^,^10^,^11^. There have been a few non-human primate studies utilizing ET-1 induced cerebral ischemia models developed in marmoset monkeys^12^,^13^. In marmosets, ET-1 was found to cause dose-dependent reductions in middle cerebral artery vessel caliber, which was followed by a gradual reperfusion. The ET-1-treated marmosets also displayed contralateral motor deficits in grip strength and the ability to retrieve food rewards as well as displayed a contralateral sensory/motor neglect towards tactile stimulation^12^. Moreover, ET-1 intracortically injected into the primary visual cortex of adult and neonatal marmosets induced posterior cerebral arterial occlusions and developed a highly reproducible and survivable model of focal ischemia^13^. These results suggested that a marmoset model of ET-1 induced ischemia has the potential to assess long-term effects of stroke and to gauge the efficacy of novel therapeutic strategies targeted to treat clinical stroke^12^,^13^. The marmoset, as a new world monkey, is limited as a model animal for human disease mechanisms in comparison to the rhesus monkey, an old world monkey. For example, the marmoset has a less intricate layer III of dlPFC pyramidal cells^14^,^15^. In addition, the rhesus monkey has many advantages to the marmoset. The cortex of the rhesus monkey is qualitatively more similar to the human cortex than any other available animal model and it has well-developed association cortices, making it a superior model to evaluate the pathology of neurological and mental illness. Taken together, the anatomy, physiology, molecular regulation and cognitive functioning of the rhesus monkey make it an optimal model animal to study stroke and brain repair.

Lesion of the motor cortex in primates is a well-established experimental model used in studying recovery from brain injury^16^,^17^,^18^,^19^,^20^,^21^,^22^ and there a number of behavioral tests that can be used to evaluate motor function in the rhesus monkey. For example, motor deficits in grip strength and in food reward retrieval can be estimated with the behavioral tests including Klüver board, vertical slot and Brinkman board tasks which evaluate dexterous hand functions, especial the digit/finger grasping ability^17^,^23^,^24^,^25^,^26^.

In the present study, the representation of the fingers and hand were mapped to the cortex of the rhesus monkey with intracortical micro-stimulation (ICMS) and then ET-1 was microinjected into the contralateral motor cortex (M1) to the dominant hand. The monkeys were trained for three manual dexterity tasks prior to the microinjection and were retested for the tasks at 3, 8, 15, 29 days after the ET-1 injection. Brain Magnetic Resonance Imaging (MRI) scans were performed 1, 7, 14 and 28 days post ischemia induction. The aim of the present work was to determine whether ET-1 induced focal ischemia in the rhesus monkey was viable and to measure the effects of the ischemia on the manual dexterity of the monkeys. The correlations between the behavioral change and the edema in the cortex were further estimated in brain MRIs in order to develop an efficient non-human primate model for studying strokes.

## Results

### Effect of ET-1 on manual dexterity in the Klüver board task

**Figure 1:**
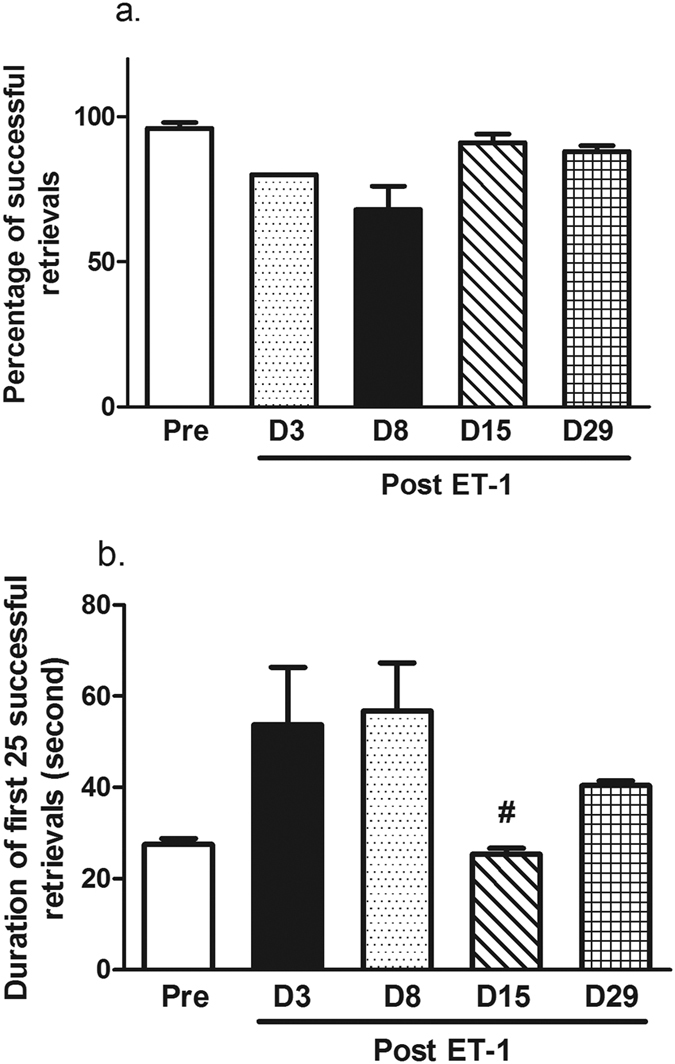
Effect of ET-1 on monkey performance in the Klüver board task. (**a**) Percentage of successful retrievals in the Klüver board task tended to decrease 8 days after the ET-1 injection. (**b**) The duration to make 25 successful retrievals. The monkeys temporally recovered 15 days after the injection when compared to the duration on D3. ^#^P < 0.05 performance on D15 vs. D3. The data are expressed as the mean ± SEM.

The percentage of successfully attempts of monkeys to retrieve the food reward from the Klüver board decreased on day 3 (D3) and day 8 (D8) after the ET-1 injection but it was not a significant difference (Fig. 1a). The duration to make 25 successful retrievals increased on D3 and D8 after the ET-1 injection (Fig. 1b). Fifteen days after the injection, the duration to make 25 retrievals returned to pre-treatment levels and was significantly reduced from D3 duration times (P = 0.042), but the D29 measurement was not totally recovered, which suggested it was a temporary recovery.

**Figure 2:**
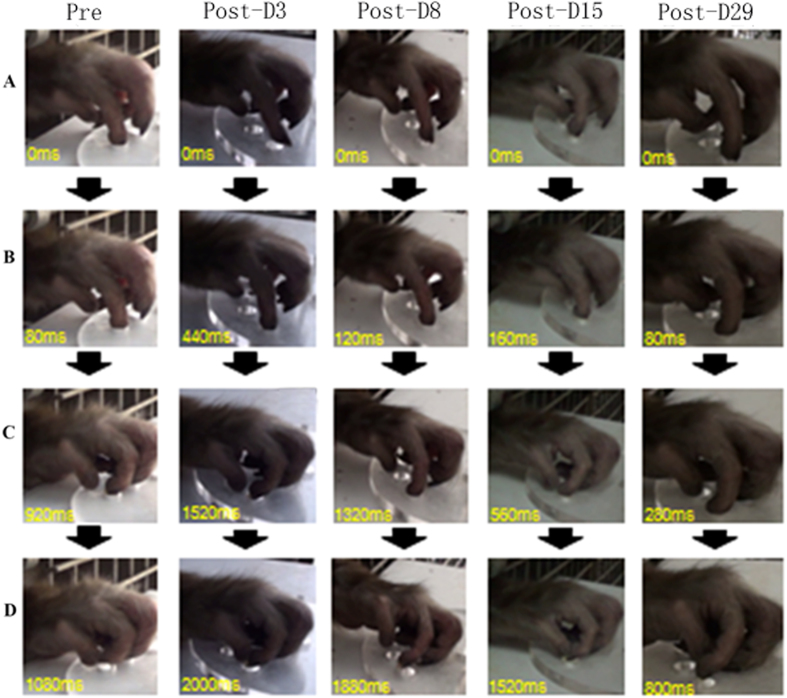
Sequence of photographs showing hand and digit movement of a trained monkey while performing the Klüver board task prior to and 3, 8, 15, 29 days after the ET-1 injection. Pre: Pre ET-1 injection, Post: post ET-1 injection. (**A–D**) presented the same pose of hand and digit at each correct movement.

Video recordings were analyzed by frames for the Klüver board task and the moment of contact between the tip of the index finger and the aperture of the well and the bottom of the well were identified, respectively. The greatest flexion of the index finger and the grasping of the food pellet were also recorded. Images of precision grips are shown in Fig. 2. The monkeys spent a longer time to generate a precision grip in retrieving the food pellet on post-injection D3 and D8. The time to retrieve the pellet returned to a lower duration at later days, suggesting that animals had some recovery from the ET-1 injection.

### Effect of ET-1 on manual dexterity in the vertical slot task

**Figure 3:**
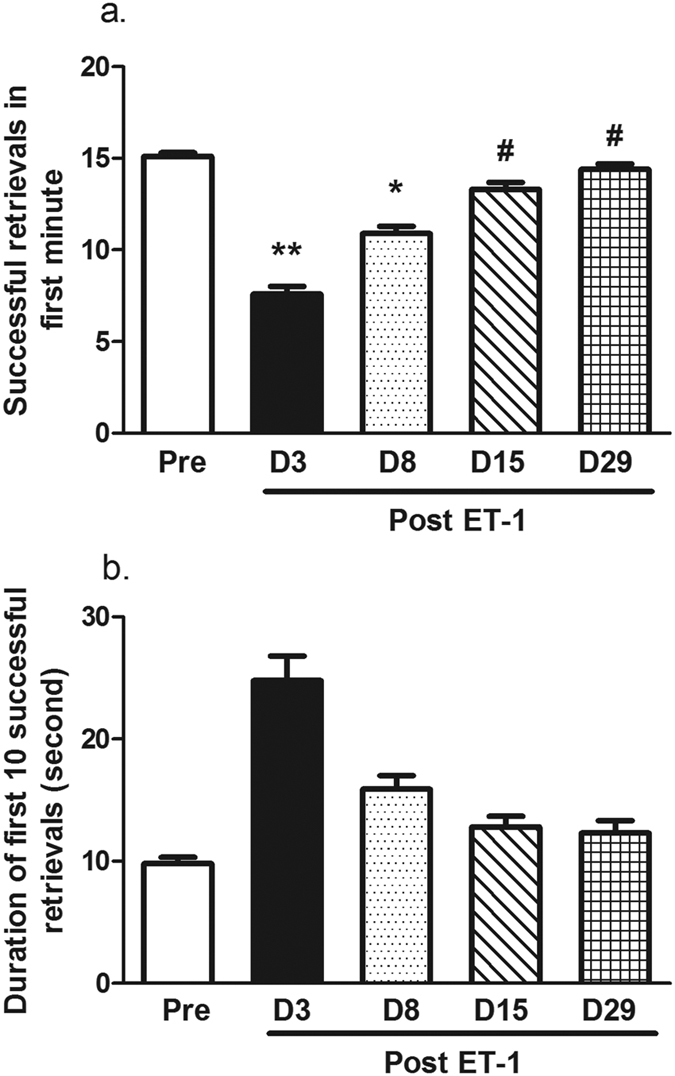
ET-1 induced performance impairments in the vertical slot task. (**a**) The number of successful retrievals in the first minute. The monkeys displayed a lower retrieval rate 3 and 8 days after ET-1 injection than before the injection. The monkeys displayed a recovered performance on days 15 and 29 after ET-1 treatment. (**b**) The time duration to make the first 10 successful retrieval movements. **P < 0.01, *P < 0.05 performance on D3 vs. Pre-injection. ^#^P < 0.05 performance on D3 vs. D15 and D29, respectively. The data are expressed as the mean ± SEM.

The number of successful food retrieval movements in the vertical slot task was decreased on D3 (P = 0.004) and D8 (P = 0.02) after ET-1 injection in comparison to performances before the injection (Fig. 3a). However, the monkeys’ ability to retrieve the food pellets had recovered at 15 days (P = 0.014) and 29 days (P = 0.016) after the ET-1 injection. This was reflected by increased successful retrievals of the food in comparison with the number of retrievals on D3.

The monkeys took a longer time to successfully retrieve 10 food pellets in the vertical slot task on day 3 after the ET-1 injection, but it was not statistically significant (Fig. 3b).

**Figure 4:**
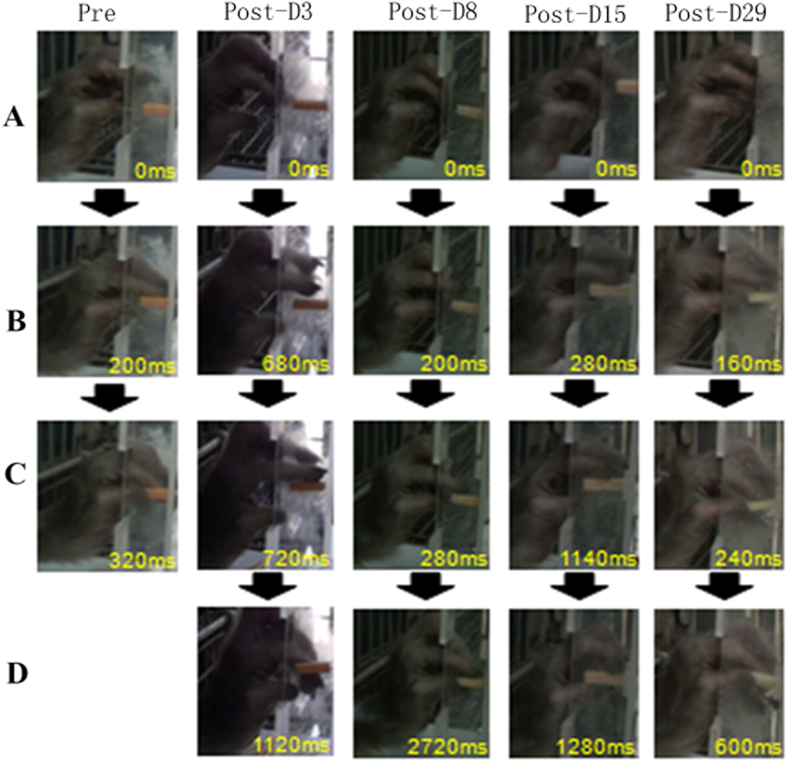
Sequence of photographs showing hand and digit movement of a trained monkey performing the vertical slot task prior to and post ET-1 injection. The monkey took a longer time to generate a precision grip while successfully retrieving the food after the ET-1 injection. Pre: Pre ET-1 injection, Post: post ET-1 injection. (**A–D**) presented the same pose of hand and digit at each correct movement.

The sequential changes in digit movement while retrieving food pellets in the vertical slot task were observed (Fig. 4). It took a longer time for the monkey to generate a precision grip on the food following the ET-1 injection.

### Effect of ET-1 on manual dexterity in the rotating Brinkman board task

**Figure 5:**
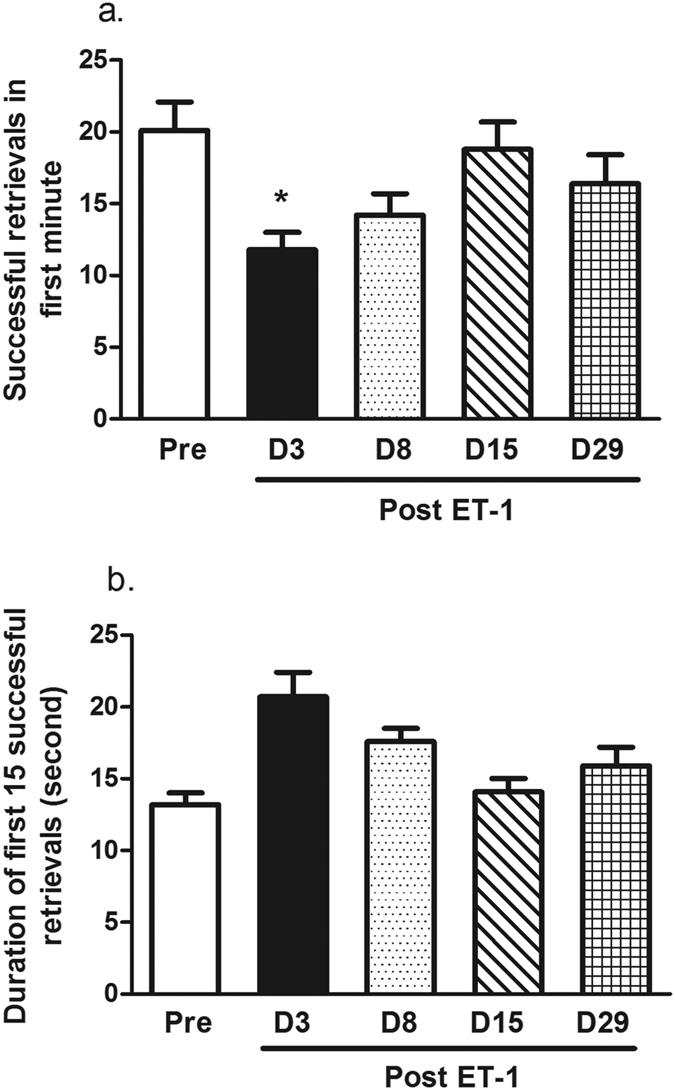
ET-1 impaired monkey manual dexterity in the rotating Brinkman board task. (**a**) Successful number of food retrievals in the first minute of the task. The retrieval of food was lower on day 3 after the ET-1 injection and gradually returned to pre-injection levels. (**b**) The duration of time to reach the first 15 successful food retrievals. There was a trend of a longer duration for the monkeys to successfully retrieve 15 food pellets after ET-1 treatment. *P < 0.05 performance on D3 vs. Pre-injection. The data are expressed as the mean ± SEM.

The number of food pellets retrieved during the first minute of the Brinkman task decreased gradually after the ET-1 injection and then gradually increased. A significant impairment in the task was found on D3 (P = 0.019; Fig. 5a). No significant difference was found in the time to make the first 15 successful retrievals, but there was a trend on D3 and D8 depicting an increased amount of time spent to complete the task (Fig. 5b).

**Figure 6:**
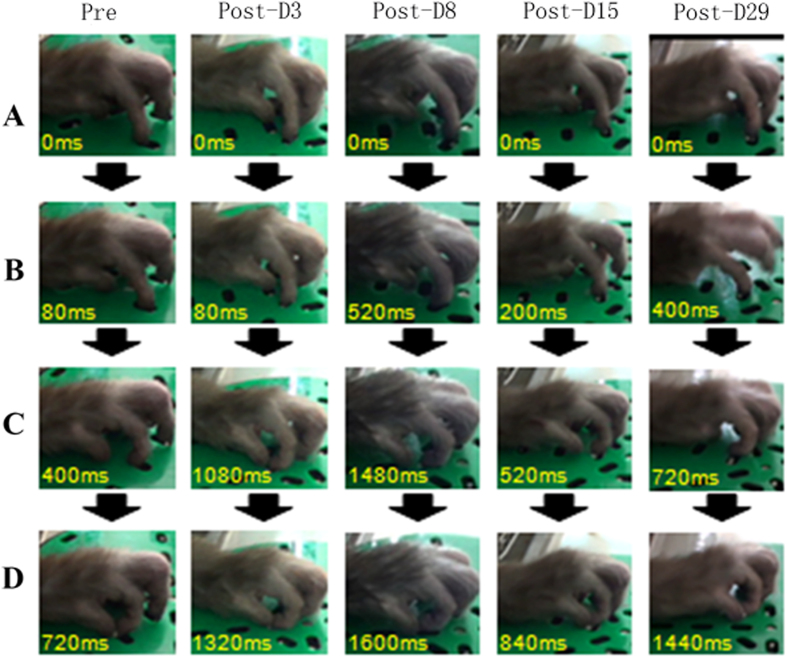
Sequence of photographs showing hand and digit movement of a trained monkey performing the rotating Brinkman board task prior to and post ET-1 injection. Pre: Pre ET-1 injection, Post: post ET-1 injection. (**A–D**) presented the same pose of hand and digit at each correct movement.

The sequential changes in digit movement while retrieving food pellets in the rotating Brinkman board task were observed (Fig. 6). It took a longer time for the monkey to generate a precision grip on the food following the ET-1 injection.

### Brain imaging analysis

To investigate the effect of ET-1 induced ischemic damage in the brain, the volume of edema and infarct were assessed with an MRI scan.

**Figure 7:**
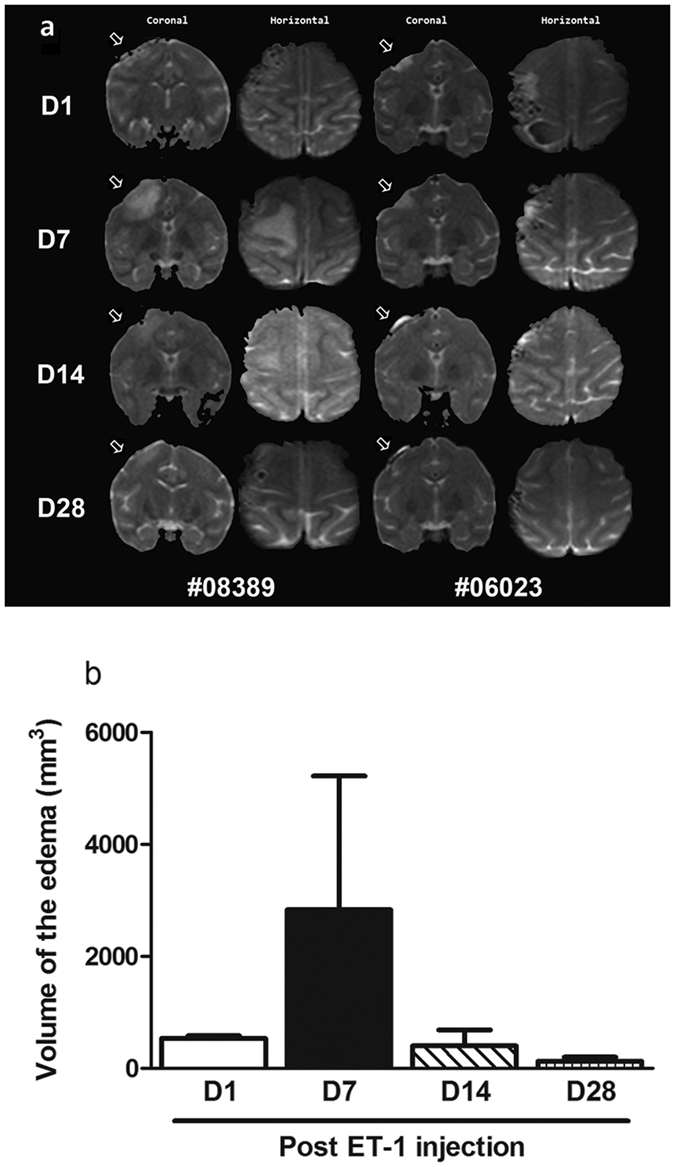
Representative MR images of two monkeys injected with ET-1 into the M1 cortex. (**a**) The infarct and edema area is denoted by hyper-intensity on T2-weighted coronal and horizontal image orientations, respectively. The hyper-intensity is marked by white arrows. (**b**) The volume of edema area in monkeys after ET-1 injection. The edema tended to increase until day 7 after the focal ischemia. The volume of the edema in the MRI reflected a severe impairment. The data are expressed as the mean ± SEM.

The ET-1 induced edema and infarct in the M1 cortex were dependent on the day after injection. The infarct was seen in the ET-1 treated group on D1. In monkey #08389, the volume of the edema was severely increased in the T2W image 7 days after the ET-1 injection. On D14, the edema volume slowly decreased and on day 28, the edema was almost unobservable in the T2W image (Fig. 7a). Similarly, the infarct volume was large in monkey #06023 on D1. The infarct became severe on D7 after the ET-1 injection. A decrease in edema was slowly seen on day 14 and the edema in monkey #06023 had been repaired on D28 in the T2W image (Fig. 7a). However, there was no significant difference between the volumes of the edema on days 7 and those calculated on days 1, 14 or 28 (Fig. 7b).

## Discussion

In the present work, ET-1 was found to induce focal ischemia and lead to impairments in the manual dexterity of rhesus monkeys assessed in the vertical slot and rotating Brinkman board tasks 3 and 8 days after the ET-1 injection. The hand dexterity of the monkeys was recovered 15–29 days after the focal ischemic stroke. Correlations were found between the behavioral impairment and the size of the edema, which was severe, 7 days after the injection with ET-1. The effects of the ET-1 induced edema were found to be ameliorated later (on D14 and D28).

The monkeys had recovered well from the surgery after one day, which was reflected by the animals’ general activity and appetite levels being similar to those levels pre-surgery. This suggested there were no side-effects from the surgery influencing the behavioral deficits on D8 and D15. These findings were consistent with previous findings in rodents^7^,^8^,^9^,^10^,^11^ and marmosets^12^,^13^. In these researches, executive function, motor function and sensorimotor behavior was impaired after intracranial ET-1 injection. In Virley *et al*.’s study, a craniotomy was made and ET-1 at different doses was applied to the M2 portion of the middle cerebral artery (MCA) in marmosets. The study found a rapid, dose-dependent and marked reduction in MCA caliber size that caused a cessation of blood flow and blanching of the cortex followed by a gradual reperfusion. ET-1– induced M2 territory lesions significantly impaired contralateral grip strength and responses to contralateral stimuli 24 hours after surgery. Virley *et al*. suggested that if a larger volume of ET-1 (at the same concentration) was used, then an M1 infarction may be more consistently produced^12^. In Teo *et al*.’s study, ET-1 was intracortically injected at four sites and at ≤ 7 sites around the posterior cerebral artery (PCA) of neonate and adult marmoset monkeys, respectively. An MRI revealed a tissue hyperintensity at the lesion site 1–7 days after the ET-1 injection followed by a tissue isointensity 14–21 days in T2-weighted images^13^. In this study, ET-1 was injected into 5 sites of the motor cortex that covered the main digit area. The location of the main digit area in the cortex of the monkeys was determined by ICSM mapping. This study found via MRI that the edema was severe 7 days after focal ischemia and the edema was ameliorated at D28. This result was consistent with Teo *et al*.’s MRI results.

A transient behavioral deficit due to the focal ischemia in the M1 area was also found in this study using three different manual dexterity tasks. This suggested that ET-1 injected into the M1 cortex, rather than into the MCA or PCA as in the two previous studies in marmosets, may be sufficient to model stroke in future studies without performing a craniotomy, a step that exposes the brain and may cause cerebral spinal fluid (CSF) leakage.

In previous studies, reperfusion was observed after ET-1 treatment was applied around the MCA or PCA in marmoset monkeys^10^,^11^ and in other studies^24^,^25^,^26^, These findings suggest the models act to mimic clinical stroke and the reperfusion after the focal ischemia in humans. In this study, tissue hyper-intensity at the lesion site was found in T2-weighted images on D7 after the ET-injection. Isointensity was found following D7. The reperfusion after ET-1 treatment was considered to be a contributing factor to this recovery, although this study did not investigate reperfusion in the monkeys. However, in immunohistochemistry studies, reactions in astrocytes, microglial cells and neurons were found to last longer than the behavioral and MRI changes (data not shown), which suggesting that the ET-1 induced ischemia might be reproducible in behavioral activities and brain imagery but that there are long-lasting effects on the brain tissue.

There are some advantages to modeling stroke with ET-1 induction in animals. First, ET-1, as a potent vasoconstrictive peptide, can be stereotaxically injected into the target areas of the brain to constrict local arterioles. Second, brain injury size can be adjusted by varying the concentration or volume of ET-1 to achieve a reproducible injury^27^. Third, ET-1 causes a dose-dependent ischemic lesion with marginal ischemic edema being observed with brain imaging scans. Moreover, MRI and Diffusion Tensor Imaging (DTI) may be applied to study the correlation between brain injury volume, trajectory and the behavioral changes of the model animals. Finally, rapid cerebral blood flow (CBF) reductions after ET-1 administration have been found in rat brain, followed by a reperfusion that occurs over several hours in different brain areas^28^,^29^,^30^. Reperfusion occurs at a much slower rate in the ET-1 induced ischemia model than with the intraluminal suture model, which better mimics clinical focal strokes and reperfusion after ischemia in humans.

It should be noted that in the current rhesus monkey model, transient rather long-lasting effects of ET-1 on behavior and on edema volume were found. This model relied on a low dose of ET-1, despite the reperfusion after ET-1 treatment, which may have contributed to the transient effects. A number of studies have shown a functional recovery following motor cortex lesions in non-human primates^21^,^22^,^23^ suggesting that another reason for the transient effects may be that monkeys have an ability to recover rapidly in the motor cortex which helps restore manual dexterity in the behavioral tasks over time. Amount studies have shown a functional recovery following motor cortex lesions in non-human primates^21^,^22^,^23^. Post-stroke axonal sprouting and rewiring and changes in dendritic branching may also contribute to the functional reorganization of the brain during recovery. In addition, repeated testing can act as training during the recovery period, leading to improved scores in the behavioral tasks. In order to avoid this confound in the present work, behavioral testing in the monkeys was performed infrequently after inducing the focal ischemic stroke.

The monkeys displayed a trend indicating an impairment in manual dexterity in the Klüver board task, but a significant difference was not obtained. This result may be due to the small subject number used in the experiment. While the small sample size is a limitation of this study because the pilot studies are often used before larger studies. In addition, individual differences in rhesus monkeys have been shown with small sample sizes in previous behavioral studies^5^,^31^,^32^ in order to provide a foundation for further work with larger sample populations.

Stroke induced infarcts commonly occur in different tissue compartments (white and gray matter) and different brain regions including the cortex, structures under the cortex and the brainstem. It has been found that the duration of impairments was longer in ET-1 induced infarcts of the white-matter than cortical infarcts in rodent^10^,^33^. This suggests that further studies injecting ET-1 into the white matter of the rhesus macaque may cause longer-lasting behavior and brain structure impairments.

In conclusion, this study confirmed the use of ET-1, a vasoconstrictor, to induce focal ischemic stroke in the rhesus monkey. This non-human primate model was survivable and provided a transient ischemia that may mimic transient focal strokes in humans. This model provides a better framework to be used in assessing the efficacy of novel therapeutic strategies targeted at repairing damages related to clinical strokes.

## Methods

### Animals

Two adult healthy rhesus monkeys (*Macaca mulatta*) weighing 8–9 kg (7–9 years old) from breeding colonies at the Kunming Institute of Zoology (KIZ) were used in these experiments. Their ID numbers were #06023 and #08389, respectively. The monkeys were housed under standard conditions (12 h light/dark cycle with light on from 07:00 to 19:00; humidity at 60% and temperature at 21 ± 2 °C). Monkeys had free access to tap water and were punctually supplied food three times a day. Experiments were performed between 8:00 and 17:00 h. All experimental procedures involving animals were performed in accordance with the guidelines for the National Care and Use of Animals approved by National Animal Research Authority of P. R. China. All efforts were made to minimize animal suffering and to reduce the number of animals required.

### Surgery and intracortical microstimulation

All surgical procedures were conducted on monkeys anesthetized with hydrochloric acidulated ketamine (He Nan Run Hong Pharmaceutical Company, 10 mg/kg, i.m.) and maintained with sodium pentobarbital (Shang Hai Westang Bio-Tech Company, 20 mg/kg, i.m.). Atropine (0.05 mg/kg, i.m.) was used to reduce salivation and other secretions. Body temperature was maintained at normal levels using a heating pad during surgery. The monkey’s head was fixed on the stereotaxic apparatus after anesthesia.

**Figure 8:**
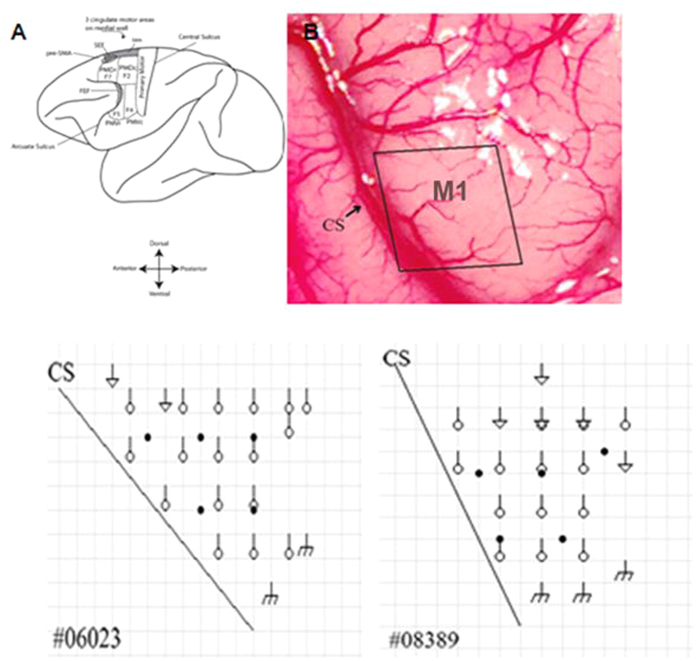
Injection areas superimposed on the ICSM maps of M1 in the two monkeys. Circles indicate ICSM induced digit movement area. Triangles indicate ICSM induced arm movement area. *m* indicates ICSM induced face movement areas. Black dots: ET-I injection sites. CS: central sulcus.

A 2 cm × 3 cm portion of skull over the precentral gyrus containing the hand representation of M1 was removed contralaterally to the dominant hand. A map of the hand representation area in primary motor cortex M1 was constructed using intracortical microstimulation (ICSM) with a HuaTuo electroacupuncture therapeutic apparatus (SDZ-V, Su Zhou Medical Instrument Co., Ltd.). Two electrode (acupuncture needles) penetrations were spaced at 2 mm intervals. One electrode was placed on the surface of the brain and the other one was placed into the cortex at a depth of 3 mm. A conventional electric stimulus (10–30 Hz, 5–25 μA) was used to evoke movement at each electrode penetration site^23^. The region of the M1 cortex corresponding to a digit within a 2 mm square of cortical surface was carefully defined by digit movement during the stimulus (Fig. 8).

After the digit representation on the cortex was mapped, ET-1 (Human and porcine, Merck. Germany) was injected at 5 sites (5 ug/10 ul/site) on the cortex that covered the main digit area. The injection was made at a depth of 3.5 mm under the surface of the cortex. The injection needle was then raised 0.5 mm after each 1/3 volume of injection. The injection was conducted under the control of an injector pump (WZ-50C6, ShangHai LanXi company) at a speed of 200 nl/min. After the surgery, the cranial opening was carefully closed and fixed to the skull by the sealed thread. The animal was allowed to recover from the anesthesia.

### Behavioral protocols

#### Pre-injection training

Monkeys were trained to perform manual dexterity tasks in the Klüver board, vertical slot task and the rotating Brinkman board^23^ before receiving an ET-1 injection. The behavior tests were started on day 3 after the surgery to allow the monkeys to receive care after the surgery that included heating. The animals were given soft food and drink and recovered from the surgery to resume normal activity after one day. *Post-injection testing:* Monkeys conducted three behavioral tasks at 3, 8, 15, 29 days after the ET-1 injection. In the testing process, the monkeys were allowed to only use their preferred hand to retrieve food pellets by placing a canvas bag sleeve on his right and non-preferred hand before the test. All experimental procedures were recorded with a HD digital video camcorder (SONY, Japan). The normal food regimen for the monkeys was withheld at breakfast and lunch time on each training and testing day.

## Training and Testing

### Klüver board task

**Figure 9:**
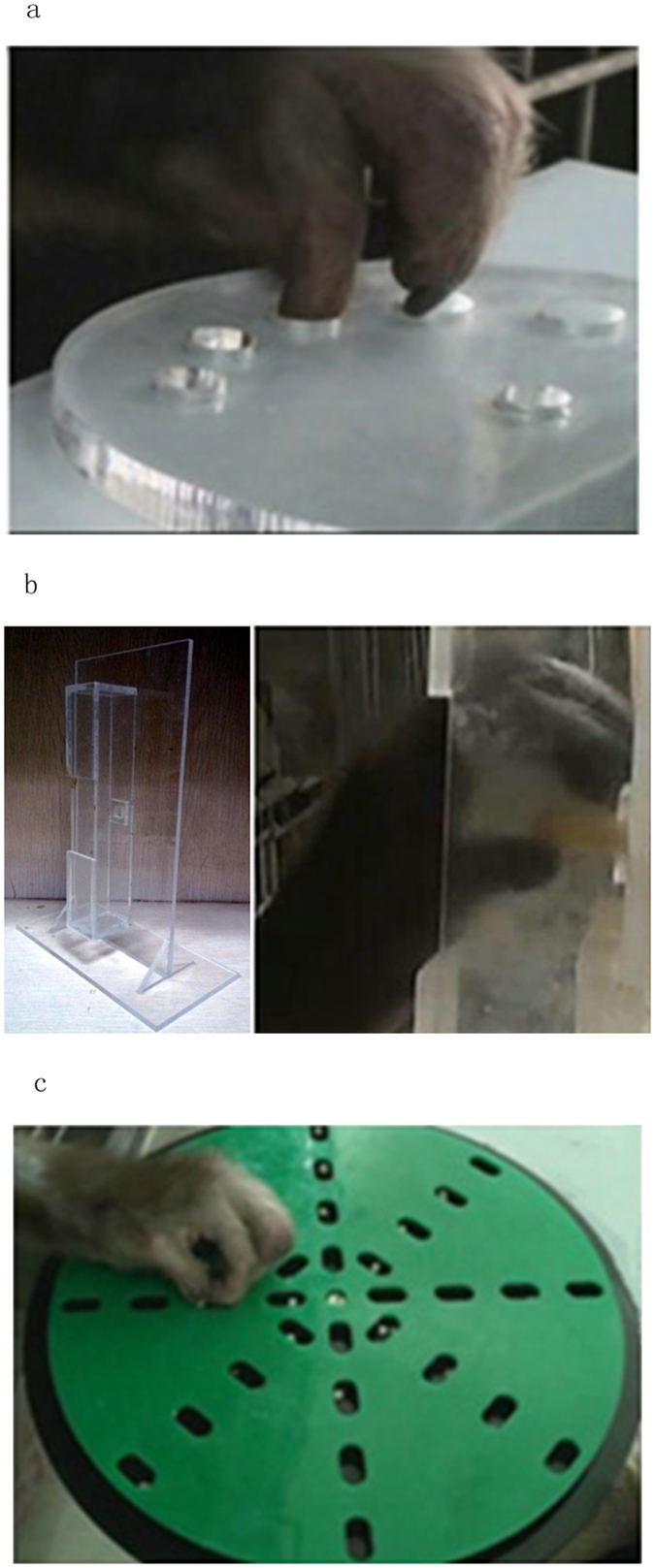
(**a**) Image of the Klüver board (**b**) Image of the vertical slot apparatus. (**c**) Image of the rotating Brickman board task.

A Klüver board containing five cylindrical wells (13 mm, 12 mm, 11 mm, 10.5 mm, and 10 mm in diameter, 7 mm in depth) was used for testing the manual dexterity of the two monkeys (Fig. 9a)^23^.

### Hand preference

A Klüver board was used to identify the monkey’s preferred and dominant hand. A small spherical food pellet (e.g. a peanut or sweet potato, 3–4 mm in diameter) was randomly placed in one of wells. Monkeys were allowed to retrieve the food for 100 trials per day and the number of times the animal reached with either hand were recorded. The preferred hand was considered to be the hand that was used to retrieve the food pellet over 80% of the trials over 2 consecutive days. All monkeys were found to prefer the left hand.

Two sessions of trainings (30 min each) were performed in the morning and in the afternoon respectively for each working day. The monkeys were trained to retrieve the food from a certain well, beginning with the largest sized well. For example, on the first training day, the largest well (13 mm in diameter, 7 mm in depth) was fixed as the place to obtain the food pellet. After a total number of 1000 successful food retrievals from the fixed well, the next smaller well was fixed as the place to obtain the food pellet on the following day. The training was ended after all the monkeys had successfully retrieved food from each well for over 1000 trials, respectively.

Before the surgery, the monkeys were tested with the Klüver board task. Twenty five food pellets were randomly placed into five different cylindrical wells, with each hole containing a total 5 pellets. The number of food pellets successfully retrieved was recorded over 30 min. The performance was scored as the percentage of correct retrieval movements and the duration to complete the first 25 successfully trials. Two sessions of testing were performed on each day; one in the morning and another in the afternoon. The testing was conducted with the same procedure on day 3, 8, 15, 29 after ET-1 injection, respectively.

### Vertical slot task

The “vertical slot task” utilizes a vertical rectangular box containing an opening in the front (2 cm × 5 cm in size, 3 cm in slot depth) and a window in the back (7.5 mm × 7.5 mm, length × width) through which the experimenter supplied the food and was used to evaluate the manual dexterity of the monkeys^23^. The food was an apple or sweet potato cut into a rectangular quadrilateral (7 mm × 7 mm × 20 mm, length × width × height). In the training and testing processes, a rectangular quadrilateral food piece was stabbed by a needle behind and placed into the center of the 7.5 mm square window where the vertical slot was located. The monkeys were allowed to retrieve the food pellets with a precision grip. After the food was successfully gripped by the monkeys, another food piece was stabbed as soon as possible by the experimenter from the other side of the apparatus. This process usually took about 1–2 seconds. The training was performed on 5 consecutive days with 30 trials for each day. The monkeys were tested for 30 trials in the testing process prior to the surgery and on day 3, 8, 15, 29 after the injection of ET-1. The number of food pellets successfully retrieved during first 1 min and the duration to successfully retrieve the first 10 food pellets were scored because the monkeys could not finish all 30 trials after the injection of ET-1 (Fig. 9b).

### Rotating Brinkman board task

A rotating brinkman board (20 cm in diameter) containing 32 oval slots (15 mm × 8 mm × 6 mm, length × width × depth) 16 vertical and 16 horizontal, distributed in four concentric rows on a Perspex board was used to measure manual dexterity^24^,^34^. A food pellet was placed in each slot before training. Food pellets were made of candy balls, sweet potato or peanut cut into a round shape about 4 mm in diameter. The board was rotating clockwise at a rate of 12 seconds per turn of the circle (Fig. 9c).

The monkeys were trained to retrieve the small food pellets from the slots using their preferred and dominant hand (left hand). Six training sessions were conducted each day with three sessions in the morning and three sessions in the afternoon. One of the three kinds of food was used in each session, respectively. The monkeys were tested with the same protocol of the training session in six sessions before the surgery as the baseline and again on day 3, 8, 15 and 29 after the injection of ET-1. The number of food pellets successfully retrieved during first 1 min and the duration to successfully retrieve the first 15 food pellets were scored because the monkeys could not retrieve all 32 trials after the injection of ET-1 (Fig. 9c).

### Brain imaging

MRI data was acquired with a PHILIPS Achieva 3.0 T MR unit at the First Affiliated Hospital of Kunming Medical University, Kunming, Yunnan, China. The brain of each monkey was scanned with the MRI on day 1, 7, 14 and 28 after the injection of ET-1. Prior to MRI scans, all monkeys were anesthetized with an i.m. injection of pentobarbital sodium. The anesthesia was stable for at least 1 hour. Three-dimensional coronal and sagittal T1, T2-weighted (T2-W) and DWI anatomical images were obtained with an inversion-recovery prepared 3-D spoiled gradient echo (SPGR) pulse sequence (TR = 14 ms; TE = 7 ms; flip angle = 8°; field of view = 160 mm; matrix = 320 × 320; number of averages = 2; final voxel resolution = 0.6 × 0.5 × 0.5 mm^3^; scan duration = 8:30 min). Brain injury volume (V) was calculated as V = ∑ ((the long axis length in the region of brain edema and infarct in coronal monolayer perpendicular × width) × section thickness).

### Statistical analysis

The data are expressed as the mean ± SEM. The statistical package SPSS 17.0 was used. Differences in behavior scores of the three tasks between the pre-injection of ET-1 and post-injection measurements and differences in brain injury volume during post-injection were assessed with paired samples T-test. Differences between treatments were considered significant when p ≤ 0.05.

## Additional Information

**How to cite this article**: PeiMin, D. *et al*. A pilot study on transient ischemic stroke induced with endothelin-1 in the rhesus monkeys. *Sci. Rep.* **7**, 45097; doi: 10.1038/srep45097 (2017).

**Publisher's note:** Springer Nature remains neutral with regard to jurisdictional claims in published maps and institutional affiliations.

## References

[b1] Di Carlo A. Human and economic burden of stroke. Age Ageing 38, 4–5, doi: afn282 (2009). 19141505 10.1093/ageing/afn282

[b2] Benowitz L. I. & Carmichael S. T. Promoting axonal rewiring to improve outcome after stroke. Neurobiol Dis 37, 259–266, doi: S0969-9961(09)00331-3 (2010). 19931616 10.1016/j.nbd.2009.11.009 PMC2818530

[b3] Starkey M. L. . Back seat driving: hindlimb corticospinal neurons assume forelimb control following ischaemic stroke. Brain : a journal of neurology 135, 3265–3281, doi: aws270 (2012). 23169918 10.1093/brain/aws270

[b4] Martins A. H. . Neuroprotective activity of (1S,2E,4R,6R,-7E,11E)-2,7,11-cembratriene-4,6-diol (4R) *in vitro* and *in vivo* in rodent models of brain ischemia. Neuroscience 291, 250–259, doi: S0306-4522(15)00143-8 (2015). 25677097 10.1016/j.neuroscience.2015.02.001 PMC4369428

[b5] Wang J. H. Non-human primate models in drug addiction deserve more attention. Dong wu xue yan jiu = Zoological research/“Dong wu xue yan jiu” bian ji wei yuan hui bian ji 35, 172–173, doi: 10.11813/j.issn.0254-5853.2014.3.172 (2014). PMC5055538 24866486

[b6] Yanagisawa M. . A novel potent vasoconstrictor peptide produced by vascular endothelial cells. Nature 332, 411–415, doi: 10.1038/332411a0 (1988). 2451132

[b7] Fuxe K. . Endothelin-1 induced lesions of the frontoparietal cortex of the rat. A possible model of focal cortical ischemia. Neuroreport 8, 2623–2629 (1997). 9261839 10.1097/00001756-199707280-00040

[b8] Abeysinghe H. C., Bokhari L., Dusting G. J. & Roulston C. L. Brain remodelling following endothelin-1 induced stroke in conscious rats. PLoS One 9, e97007, doi: 10.1371/journal.pone.0097007PONE-D-13-54235 (2014). 24809543 PMC4029108

[b9] Cordova C. A., Jackson D., Langdon K. D., Hewlett K. A. & Corbett D. Impaired executive function following ischemic stroke in the rat medial prefrontal cortex. Behav Brain Res 258, 106–111, doi: S0166-4328(13)00630-X (2014). 24144544 10.1016/j.bbr.2013.10.022

[b10] Blasi F., Whalen M. J. & Ayata C. Lasting pure-motor deficits after focal posterior internal capsule white-matter infarcts in rats. Journal of cerebral blood flow and metabolism : official journal of the International Society of Cerebral Blood Flow and Metabolism 35, 977–984, doi: jcbfm20157 (2015). 10.1038/jcbfm.2015.7 PMC4640262 25649992

[b11] Tennant K. A. & Jones T. A. Sensorimotor behavioral effects of endothelin-1 induced small cortical infarcts in C57BL/6 mice. Journal of neuroscience methods 181, 18–26, doi: S0165-0270(09)00194-0 (2009). 19383512 10.1016/j.jneumeth.2009.04.009 PMC6667280

[b12] Virley D. . A new primate model of focal stroke: endothelin-1-induced middle cerebral artery occlusion and reperfusion in the common marmoset. Journal of cerebral blood flow and metabolism : official journal of the International Society of Cerebral Blood Flow and Metabolism 24, 24–41, doi: 10.1097/01.WCB.0000095801.98378.4A (2004). 14688614

[b13] Teo L. & Bourne J. A. A reproducible and translatable model of focal ischemia in the visual cortex of infant and adult marmoset monkeys. Brain Pathol 24, 459–474, doi: 10.1111/bpa.12129 (2014). 25469561 PMC8029183

[b14] Elston G. N. . Specializations of the granular prefrontal cortex of primates: implications for cognitive processing. Anat Rec A Discov Mol Cell Evol Biol 288, 26–35, doi: 10.1002/ar.a.20278 (2006). 16342214

[b15] Elston G. N. Cortex, cognition and the cell: new insights into the pyramidal neuron and prefrontal function. Cereb Cortex 13, 1124–1138 (2003). 14576205 10.1093/cercor/bhg093

[b16] Pribram K. H., Kruger L., Robinson F. & Berman A. J. The effects of precentral lesions on the behavior of monkeys. Yale J Biol Med 28, 428–443 (1955). 13291869 PMC2603400

[b17] Brinkman C. Lesions in supplementary motor area interfere with a monkey’s performance of a bimanual coordination task. Neurosci Lett 27, 267–270 (1981). 7329632 10.1016/0304-3940(81)90441-9

[b18] Rouiller E. M. . Dexterity in adult monkeys following early lesion of the motor cortical hand area: the role of cortex adjacent to the lesion. Eur J Neurosci 10, 729–740 (1998). 9749734 10.1046/j.1460-9568.1998.00075.x

[b19] Eisner-Janowicz I. . Early and late changes in the distal forelimb representation of the supplementary motor area after injury to frontal motor areas in the squirrel monkey. J Neurophysiol 100, 1498–1512, doi: 90447.2008 (2008). 18596180 10.1152/jn.90447.2008 PMC2544457

[b20] Darling W. G. . Volumetric effects of motor cortex injury on recovery of dexterous movements. Exp Neurol 220, 90–108, doi: S0014-4886(09)00313-6 (2009). 19679127 10.1016/j.expneurol.2009.07.034 PMC2778269

[b21] Higo N. Training-induced recovery of manual dexterity after a lesion in the motor cortex. Keio J Med 59, 4–9 (2010). 20375652 10.2302/kjm.59.4

[b22] Darling W. G., Pizzimenti M. A. & Morecraft R. J. Functional recovery following motor cortex lesions in non-human primates: experimental implications for human stroke patients. J Integr Neurosci 10, 353–384, doi: S0219635211002737 (2011). 21960307 10.1142/S0219635211002737 PMC3689229

[b23] Murata Y. . Effects of motor training on the recovery of manual dexterity after primary motor cortex lesion in macaque monkeys. J Neurophysiol 99, 773–786, doi: 01001.2007 (2008). 18094104 10.1152/jn.01001.2007

[b24] Schmidlin E. . Behavioral assessment of manual dexterity in non-human primates. Journal of visualized experiments : JoVE, doi: 3258 (2011). 10.3791/3258 PMC3308590 22105161

[b25] Milliken G. W., Plautz E. J. & Nudo R. J. Distal forelimb representations in primary motor cortex are redistributed after forelimb restriction: a longitudinal study in adult squirrel monkeys. J Neurophysiol 109, 1268–1282, doi: jn.00044.2012 (2013). 23236004 10.1152/jn.00044.2012 PMC3602839

[b26] Brinkman C. Supplementary motor area of the monkey’s cerebral cortex: short- and long-term deficits after unilateral ablation and the effects of subsequent callosal section. The Journal of neuroscience : the official journal of the Society for Neuroscience 4, 918–929 (1984). 6716131 10.1523/JNEUROSCI.04-04-00918.1984 PMC6564786

[b27] Murphy T. H. & Corbett D. Plasticity during stroke recovery: from synapse to behaviour. Nature reviews. Neuroscience 10, 861–872, doi: nrn2735 (2009). 19888284 10.1038/nrn2735

[b28] Macrae I. M., Robinson M. J., Graham D. I., Reid J. L. & McCulloch J. Endothelin-1-induced reductions in cerebral blood flow: dose dependency, time course, and neuropathological consequences. Journal of cerebral blood flow and metabolism : official journal of the International Society of Cerebral Blood Flow and Metabolism 13, 276–284, doi: 10.1038/jcbfm.1993.34 (1993). 8436619

[b29] Biernaskie J., Corbett D., Peeling J., Wells J. & Lei H. A serial MR study of cerebral blood flow changes and lesion development following endothelin-1-induced ischemia in rats. Magn Reson Med 46, 827–830, doi: 10.1002/mrm.1263 (2001). 11590661

[b30] Sharkey J., Ritchie I. M. & Kelly P. A. Perivascular microapplication of endothelin-1: a new model of focal cerebral ischaemia in the rat. Journal of cerebral blood flow and metabolism : official journal of the International Society of Cerebral Blood Flow and Metabolism 13, 865–871, doi: 10.1038/jcbfm.1993.108 (1993). 8360292

[b31] Wang J. H. . Interactive effects of morphine and dopaminergic compounds on spatial working memory in rhesus monkeys. Neuroscience bulletin 29, 37–46, doi: 10.1007/s12264-013-1305-3 (2013). 23361521 PMC5561866

[b32] Wu X. . Morphine-induced conditioned place preference in rhesus monkeys: Resistance to inactivation of insula and extinction. Neurobiology of learning and memory 131, 192–200, doi: S1074-7427(16)30029-6 (2016). 27101734 10.1016/j.nlm.2016.04.005

[b33] Blasi F. . Recognition memory impairments after subcortical white matter stroke in mice. Stroke; a journal of cerebral circulation 45, 1468–1473, doi: STROKEAHA.114.005324 (2014). 10.1161/STROKEAHA.114.005324 24723319

[b34] Kaeser M. . Effects of unilateral motor cortex lesion on ipsilesional hand’s reach and grasp performance in monkeys: relationship with recovery in the contralesional hand. J Neurophysiol 103, 1630–1645, doi: 00459.2009 (2010). 20071636 10.1152/jn.00459.2009

